# Organic radical materials in biomedical applications: State of the art and perspectives

**DOI:** 10.1002/EXP.20210264

**Published:** 2022-03-17

**Authors:** Xiao Cui, Zhen Zhang, Yuliang Yang, Shengliang Li, Chun‐Sing Lee

**Affiliations:** ^1^ Department of Chemistry Institution Center of Super‐Diamond and Advanced Films (COSDAF) City University of Hong Kong Kowloon Hong Kong SAR China; ^2^ College of Pharmaceutical Sciences Soochow University Suzhou China

**Keywords:** bioimaging, open‐shell, photodynamic therapy, photothermal therapy, radical

## Abstract

Owing to their unique chemical reactivities and paramagnetism, organic radicals with unpaired electrons have found widespread exploration in physical, chemical, and biological fields. However, most radicals are too short‐lived to be separated and only a few of them can maintain stable radical forms via stereochemical strategies. How to utilize these raw radicals for developing stable radical‐containing materials have long been a research hotspot for many years. This perspective introduces fundamental characteristics of organic radical materials and highlights their applications in biomedical fields, particularly for bioimaging, biosensing, and photo‐triggered therapies. Molecular design of these radical materials is considered with reference to their outstanding imaging and therapeutic performances. Various challenges currently limiting the wide applications of these organic radical materials and their future development are also discussed.

## Graphical abstract

Organic radicals usually show unique chemical reactivities and paramagnetism, which have aroused wide concern in physical, chemical, and biological fields for many years. Here we summarized the biomedical applications of radical‐containing materials in imaging/sensing (e.g., EPR imaging, spin labeling, magnetic resonance imaging, and fluorescence imaging ) and photo‐triggered therapies (photodynamic therapy and photothermal therapy). We introduced the fundamental characteristics of representative organic radical materials and highlighted their design principle and working mechanism with reference to these biomedical uses.

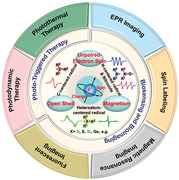

## 1 INTRODUCTION

Radicals are typically open‐shell species, in which there are one or more unpaired electrons.^[^ ^1^ ^]^ For example, some oxygen‐centered radicals such as hydroxyl radicals (•OH) and superoxide radicals (O_2_^•−^) are involved in many biological processes. Even a low concentration of radicals can play crucial roles in cell proliferation and invading organisms in disease‐resistance. Meanwhile, overaccumulation of radicals generally induces oxidative damage to biomolecules such as nucleic acid, proteins, and lipid, which closely correlates with many diseases.^[^ ^2^ ^]^ However, these oxygen‐centered radicals are typically unstable and short‐lived (10^–9 ^s) because of their incompletely satisfied valency and intrinsically high energy. In 1900, a stable organic carbon‐centered radical triphenylmethyl was firstly discovered by Gomberg.^[^ ^3^ ^]^ This is recognized as a landmark, and it also lays the foundation for developing other stable radical materials. Since then, researchers realized that while most radicals are open‐shelled and chemically active, relatively stable active centers can be maintained if they are sterically protected.^[^ ^4^ ^]^ Moreover, the unpaired electrons endow radicals with unique magnetic, optical, electronic, and redox properties, which can be exploited for applications in spin probes, optoelectronic devices, and energy storage.^[^ ^5^ ^]^ On the other hand, stability is highly indispensable for their broad applications. Spin density delocalization (e.g., by covalently linking π‐conjugates, electron‐rich or deficient moieties, heteroatoms, or noncovalently using hydrogen band or electrostatic interaction) and steric protection approaches (e.g., by noncovalently using host‐guest supramolecular chemistry, bulky substituents) have been exploited to obtain long‐lived radicals that can last for few hours/days even few months. Compared to non‐covalent methods, covalent methods generally need complicated or harsh synthesis conditions. Moreover, it should be noted that radicals obtained via non‐covalent methods can be remotely controlled by external stimuli, suggesting their potential for designing smart systems.^[^ ^1^, ^4^, ^6^ ^]^

As mentioned, the existence of unpaired electrons in the open‐shelled structures endows radicals with superior spin, magnetics, optics, and redox over traditional closed‐shell molecules. Furthermore, via rational molecular design, radical materials can provide unusual properties not found in most closed‐shelled molecules. Several previous literatures have already summarized synthesis methods, stability tuning strategies and spin, optoelectronic, energy applications of organic radical materials.^[^ ^1^, ^4^, ^6^, ^7^ ^]^ However, so far there are little reports on their biomedical applications. The aim herein is to highlight the recent achievements of radicals in materials science, biochemistry, biology, and biophysics, and further offer a connection between different disciplines.

## 2 CLASSIFICATIONS OF ORGANIC RADICAL MATERIALS

**FIGURE 1:**
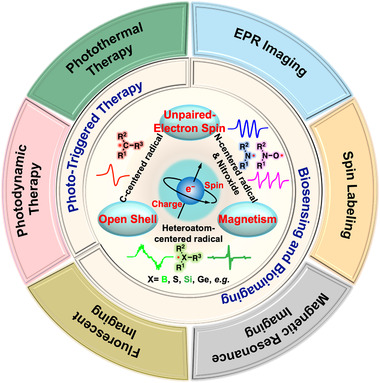
Biomedical applications of organic radical materials

**FIGURE 2:**
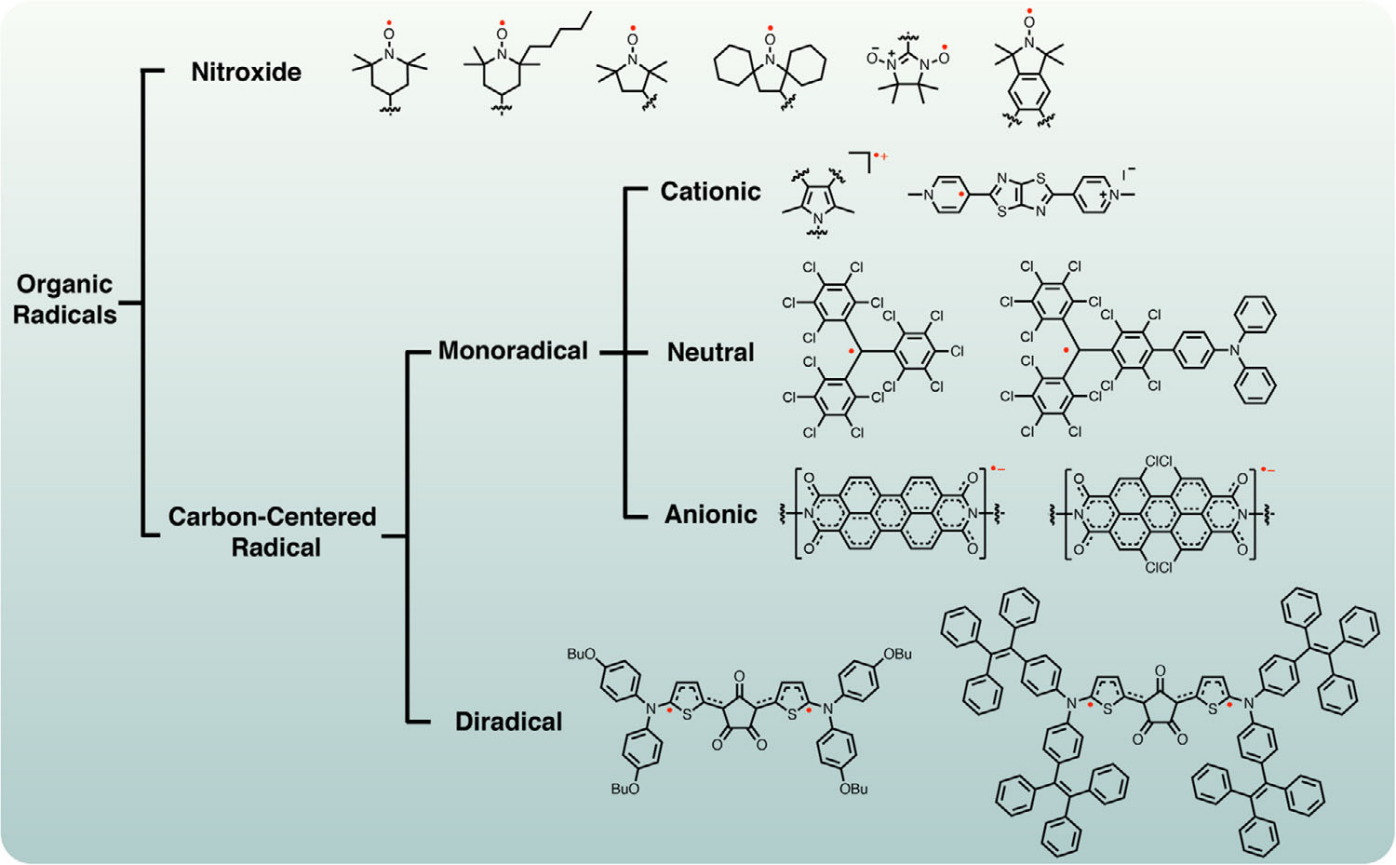
The classifications of organic radicals

Most reported organic radical materials have their active centers on carbon,^[^ ^8^ ^]^ nitroxide,^[^ ^9^ ^]^ silicon,^[^ ^10^ ^]^ germanium,^[^ ^11^ ^]^ beryllium,^[^ ^12^ ^]^ and boryl^[^ ^13^ ^]^ atoms/groups. They typically demonstrated center‐atom dependent exclusive ‘fingerprint’ signals in electron paramagnetic resonance (EPR) spectroscopy, as shown in Figure 1. Classifications and molecular structures of organic radicals mentioned in this perspective are listed in Figure 2. For example, 2,2,6,6‐tetramethyl‐piperidinyloxy (TEMPO) is a commercially available nitroxide monoradical for half a century where an unpaired electron is distributed between a nitrogen and an oxygen atom. TEMPO has been extensively employed in vitro and in vivo because of its good water solubility and stability, as well as good membrane permeability.^[^ ^7^ ^]^ Besides the difference of active centers, neutral, cationic, and anionic radicals can be easily distinguished according to charge valence. Once a neutral radical loses or gains an electron, they will readily become cationic or anionic. In view of the number and the exchange interaction of unpaired electrons, there are monoradical, diradical (or biradical), polyradicals. Molecules containing one unpaired electron refer to monoradicals with a spin quantum number of *S* = 1/2. The spin multiplicity (2S+1) of monoradicals has a doublet ground state, which is totally different from singlet state as ground state in a closed‐shell molecule. In a diradical, there are two unpaired electrons that either interacted or are independent. For diradicals with well‐separated radicals, they behave as two independent doublets. On the other hand, in a diradical with interacting electrons, the ground state can be a singlet (*S* = 0, spin multiplicity = 1) or a triplet spin state (*S* = 1, spin multiplicity = 3) in an external magnetic field.^[^ ^1^, ^4^, ^14^ ^]^

## 3 BIOIMAGING AND BIOSENSING

### 3.1 Magnetic resonance imaging (MRI)

MRI is an important imaging tool owning to its merits of high penetration depth, three‐dimensioned spatial resolution, less invasiveness, and excitation source‐free. MRI contrast agents can in situ reduce the relaxation time of proton (^1^H) either from longitudinal relaxation (T_1_, spin‐lattice) or transverse relaxation (T_2_, spin‐spin). Gadolinium (Gd) complexes, manganese (Mn) complexes, iron (Fe)‐oxides are most used contrast agents for T_1_‐weighted and/or T_2_‐weighted imaging.^[^ ^15^ ^]^ Gadolinium(III)‐based contrast agents (GBCAs) in the clinic are one of the most successful contrast agents. However, these metal‐based contrast agents can increase the risk of patients’ exposure to toxicity if residual metal ions accumulated in organs and tissues.^[^ ^16^ ^]^ As owner of unpaired electrons, organic radicals are often featured with paramagnetic or superparamagnetic doublet properties, which make them qualified as MRI contrast agents. Moreover, most organic radicals are metal‐free species with superior biocompatibility, thus offering a safer alternative.

**FIGURE 3:**
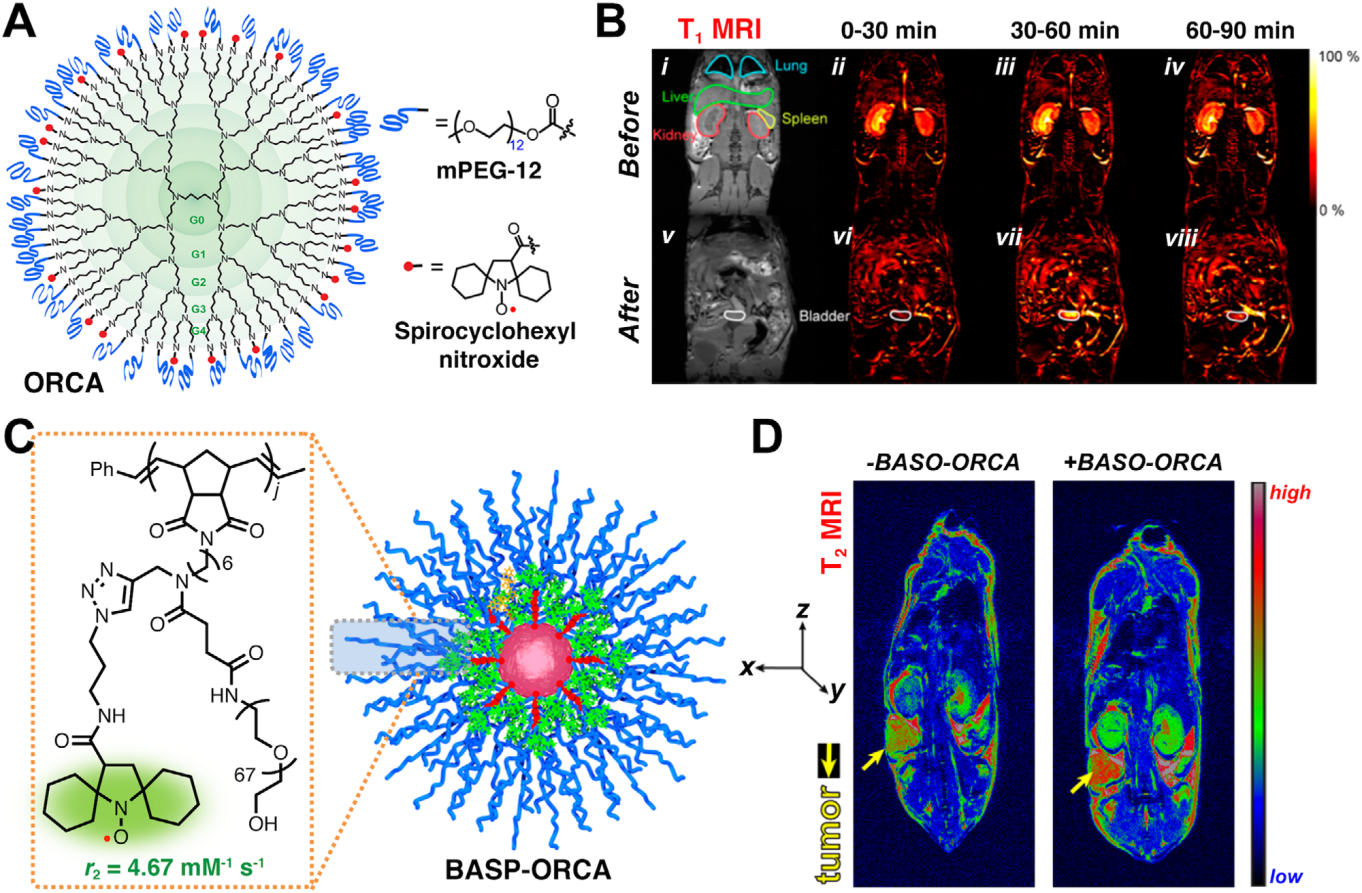
(A) Organic radical contrast agent (ORCA) and chemical structures of mPEG‐12 and spirocyclohexyl nitroxide. (B) (i,v) T1‐weighted spoiled gradient recalled echo MRI of mouse before and after injection of ORCA. MRI images obtained in different periods (ii,vi) 0–30 min, (iii,vii) 30–60 min and (iv,viii) 60–90 min. Reproduced with permission.^[^ ^17^ ^]^ Copyright 2012, American Chemical Society. (C) BASP‐ORCAs with brush‐arm star shape and chemical structure of nitroxide. (D) T2‐weighted MRI images before and after BASP‐ORCA injection. Reproduced with permission.^[^ ^19^ ^]^ Copyright 2017, American Chemical Society

Rajca et al. reported a nitroxide organic contrast agent (ORCA), which is fabricated by conjugating nitroxide radicals and poly(ethylene glycol) (PEG) chains to the surface of polypropylenimine dendrimer, as shown in Figure 3A.^[^ ^17^ ^]^ The unique dendrimer design endows it with reduction‐resistance and higher relaxivity than linear structures. The introduction of PEG is for better water‐dispersibility (0.5 g/ml). The relaxivity value (r_1_) of ORCA is calculated to be 5 mM^–1^ s^–1^, highly superior to the commonly used 3‐carboxy‐2,2,5,5‐tetramethyl‐l‐pyrrolidinyloxy nitroxide (3‐CP, 0.14 mM^–1^ s^–1^). To study whether ORCA is qualified as a practical MRI contrast agent in vivo, T_1_‐weighted imaging of mice was conducted. The results showed that ORCA has a long lifetime in vivo and can selectively image mice kidney for over 1 h with enhanced MRI signal (Figure 3B). No obvious uptake was observed in liver and spleen, and the lightening up of lung resulted from the enhancement of the vasculature rather than the uptake of ORCA in lung.

Johnson et al. developed a series of branched‐brush shape nitroxides as MRI contrast agents. In 2014, they designed a branched‐bottlebrush polymer to achieve both MRI and fluorescence imaging.^[^ ^18^ ^]^ In a later report, they further demonstrated a series of brush‐arm star polymer organic radical contrast agents (BASP‐ORCAs).^[^ ^19^ ^]^ BASP‐ORCAs have a polyacetal core and a PEG shell with abundant nitroxide groups surface bonding (Figure 3C). Among BASP‐ORCAs, BASP‐ORCA1 simultaneously guarantees good water solubility (50 mg/ml) and demonstrates a high transverse (r_2_) relaxivity of 4.67 mM^–1^ s^–1^, which is far higher than that of 3‐CP (0.17 mM^–1^ s^–1^). For in vivo imaging, obvious contrast differences can be found in the presence and absence of BASP‐ORCA1 injection (Figure 3D, yellow arrows). Overall, BASP‐ORCA1 shows a high transverse relaxivity, long‐term in vivo stability, good solubility, and low toxicity, suggesting that nitroxides are potential alternatives to metal‐based MRI contrast agents.

### 3.2 EPR imaging

**FIGURE 4:**
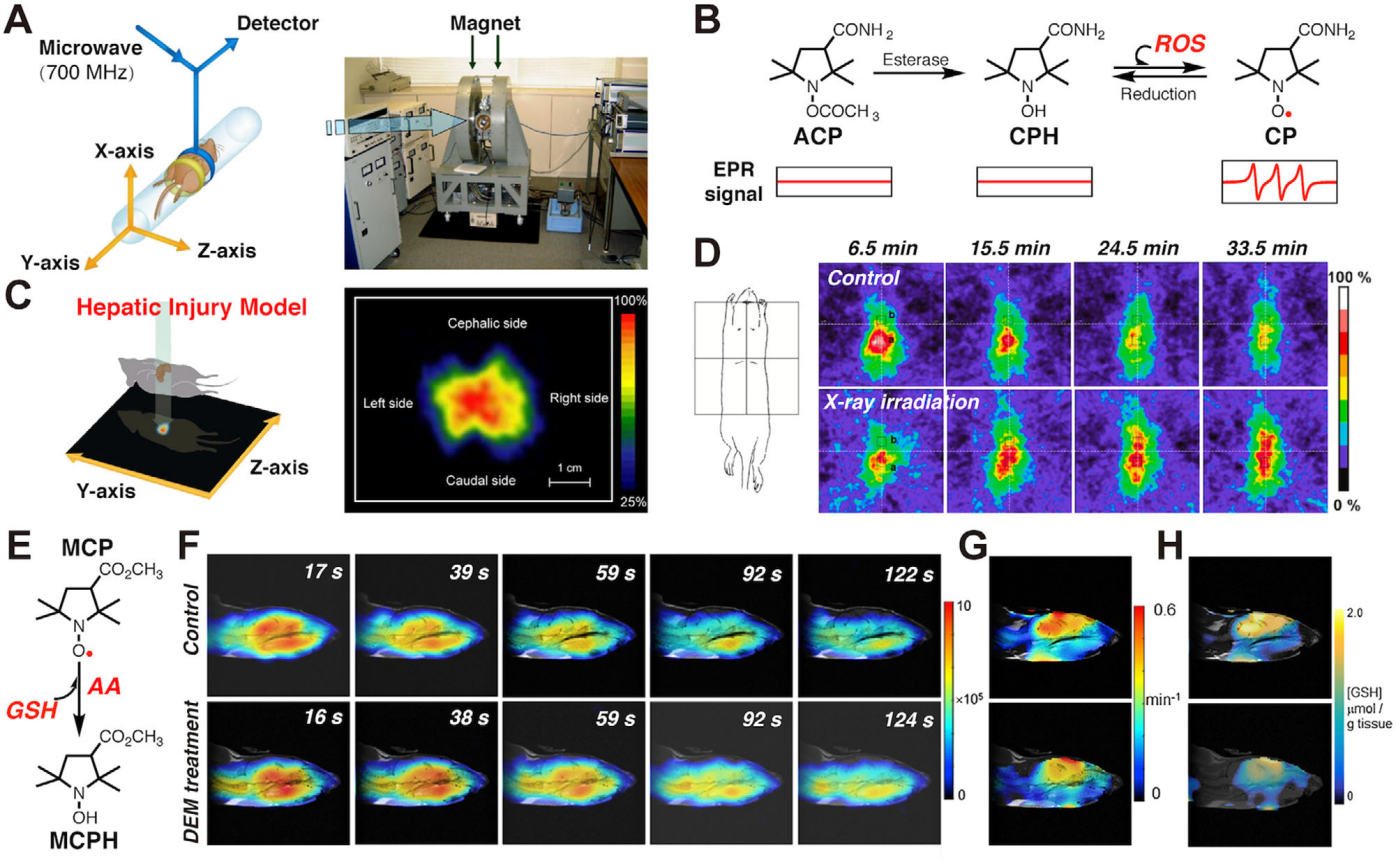
(A) Construction of an EPR imaging system. (B) Structures of ACP, CPH, CP, and their conversions. (C) In vivo EPR imaging of ROS distribution in hepatic injury models. Reproduced with permission.^[^ ^21^ ^]^ Copyright 2016, John Wiley and Sons. (D) Time‐dependent EPR images of CP in mice before or after X‐ray irradiation. Reproduced with permission.^[^ ^22^ ^]^ Copyright 2020, Elsevier. (E) Molecular structures of MCP and MCPH and their conversions. (F) EPR imaging of MCP without (control) and with DEM treatment. (G) Corresponding redox map from (F). (H) The map of GSH level. Reproduced with permission.^[^ ^23^ ^]^ Copyright 2019, Elsevier

Apart from MRI, the special magnetic properties of nitroxides can also be readily analyzed by EPR spectroscopy.^[^ ^20^ ^]^ Under redox environment, nitroxides can convert reversibly with their non‐radical (hydroxylamine) forms. While nitroxide radicals generally enable strong EPR signals, their non‐radical forms give little EPR signal. In a healthy tissue or organ, reductants, such as glutathione (GSH) and ascorbic acid (AA) exist in organs or tissues are in a balance with ROS to maintain a redox homeostasis. Overaccumulation of ROS within organs or tissues would cause serious oxidative damages to biomolecules leading to various diseases including Alzheimer, cancer, diabetes mellitus, cardiovascular disease, liver injury, inflammation, neurodegenerative injury, etc. Thus, by monitoring interconversion between nitroxides and their reduced hydroxylamine forms via EPR, biological redox status can be clearly mapped. Togashi et al. reported a low frequency EPR system for imaging in vivo oxidative free radicals.^[^ ^21^ ^]^ This device consisted of a main magnet, a pair of field gradient coils, a 700‐MHz microwave EPR unit, and a detector (Figure 4A). The EPR imaging system makes it possible to spatiotemporally visualize localizations and levels of ROS within living bodies. They used 1‐acetoxy‐3‐carbamoyl 2,2,5,5‐tetramethylpyrroline (ACP) as a spin probe (Figure 4B). The acyl group of ACP can be easily removed by both in vitro and in vivo esterase to yield hydroxylamine form (CPH). CPH with reducing ability can react with overproduced in vivo free radicals to yield the corresponding nitroxide (CP), which can provide EPR signal and enable ROS visualization in vivo. In a stimulated hepatic injury model induced by lipopolysaccharide (LPS), two‐dimensioned EPR image (*Z* and *Y* axes) reveals that ROS mainly distributed in the liver (Figure 4C). This work significantly promotes the development of in vivo ROS imaging by EPR. Shortly afterward, Takeshita et al. used the same spin probe and approach to study a liver damage model induced by X‐ray irradiation.^[^ ^22^ ^]^ Figure 4D shows EPR images of in vivo CP before (control) and after X‐ray irradiation (7.5 Gy). Compared to the control group, in which no distinguished EPR signals can be selectively observed in liver, significantly enhanced EPR signals were captured in X‐ray treated liver and the intensity of EPR signal is well maintained after ACP injection for 33.5 min (Figure 4D).

Fujii et al. then used a nitroxide 3‐methoxycarbonyl‐2,2,5,5‐tetramethylpiperidine‐1‐oxyl (MCP) to spatially monitor the redox status from GSH‐depleted mouse brains, as shown in Figure 4E.^[^ ^23^ ^]^ MCP can offer strong EPR signal, and the signal gradually decreased after its reduction into hydroxylamine form (MCPH) by GSH and AA. To build a GSH‐depleted mouse brain model, mice were treated with diethyl maleate (DEM). Temporal EPR/MRI images show that the reduction rate of MCP in presence of DEM decreased compared to that of control group (Figure 4F). Based on the EPR/MRI images and pharmacokinetics, MCP reduction rates (Figure 4G) in the control group and the DEM‐treated group were calculated. Moreover, maps of GSH distribution were obtained according to the reduction rates (Figure 4H). In control group, GSH mainly located in the cerebellum of brain. By contrast, the level of GSH in these regions obviously decreased in the DEM‐treatment mice.

### 3.3 Spin labeling

**FIGURE 5:**
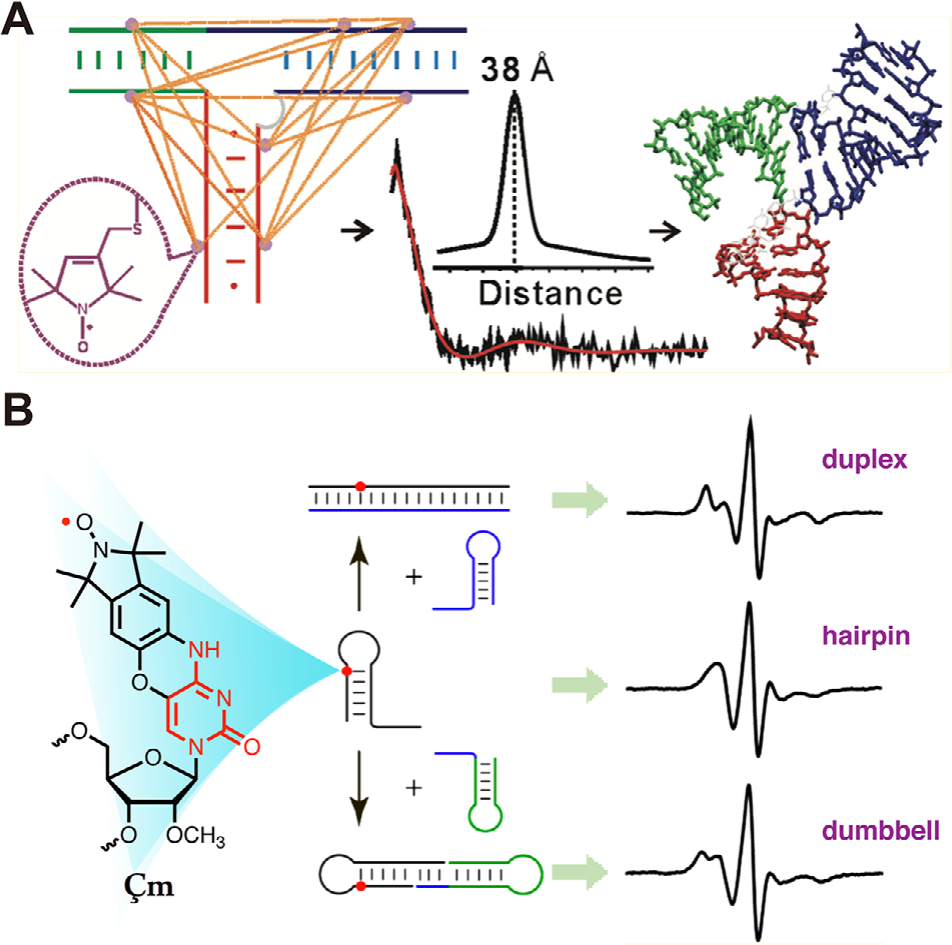
(A) Schematic illustration of mapping conformation of RNA using nitroxide spin labels. Reproduced with permission.^[^ ^25^ ^]^ Copyright 2012, American Chemical Society. (B) Chemical structures of Çm and CW‐EPR spectra of Çm spin‐labeled different RNAs. Reproduced with permission.^[^ ^26^ ^]^ Copyright 2012, American Chemical Society

Spin properties of organic radicals also enable them to label biomolecules such as nucleic acids and proteins. By inserting these radical at specific positions of biomolecules, known as “in site‐directed spin labeling (SDSL)”, their structures and dynamics can be studied.^[^ ^24^ ^]^ By using pulsed EPR techniques, the inter‐distance between radicals can also be observed through studying electron spin dipolar coupling. Recently, to observe the conformation of a packaging RNA (pRNA) dimer, Qin et al. attached stable nitroxide groups to eight specific sites of the pRNA, which is an important packing motor (Figure 5A).^[^ ^25^ ^]^ Double electron‐electron resonance spectroscopy was used for studying the distance (20–80 Å) between a pair of nitroxides in solution. The results reveal 17 inter‐nitroxide distances spanning the three‐way junction.

Sigurdsson et al. synthesized nucleoside Çm containing a nitroxide connected with the exocyclic amino group of a nucleobase (Çm), which was incorporated into different RNA secondary structures (Figure 5B).^[^ ^26^ ^]^ It was shown with EPR spectroscopy that Çm effectively offers local conditions for the labeling site and provided information on the global RNA structure (hairpin versus duplex or dumbbell). Çm‐labeled hairpin (10aÇm) exhibits three resolved hyperfine lines. The spectrum of Çm‐labeled duplex is broadened because of increased size and decreased rotational correlation times. To further reveal RNA secondary structures with Çm, they further revealed the generation of dumbbell structures.

### 3.4 Fluorescence sensing

**FIGURE 6:**
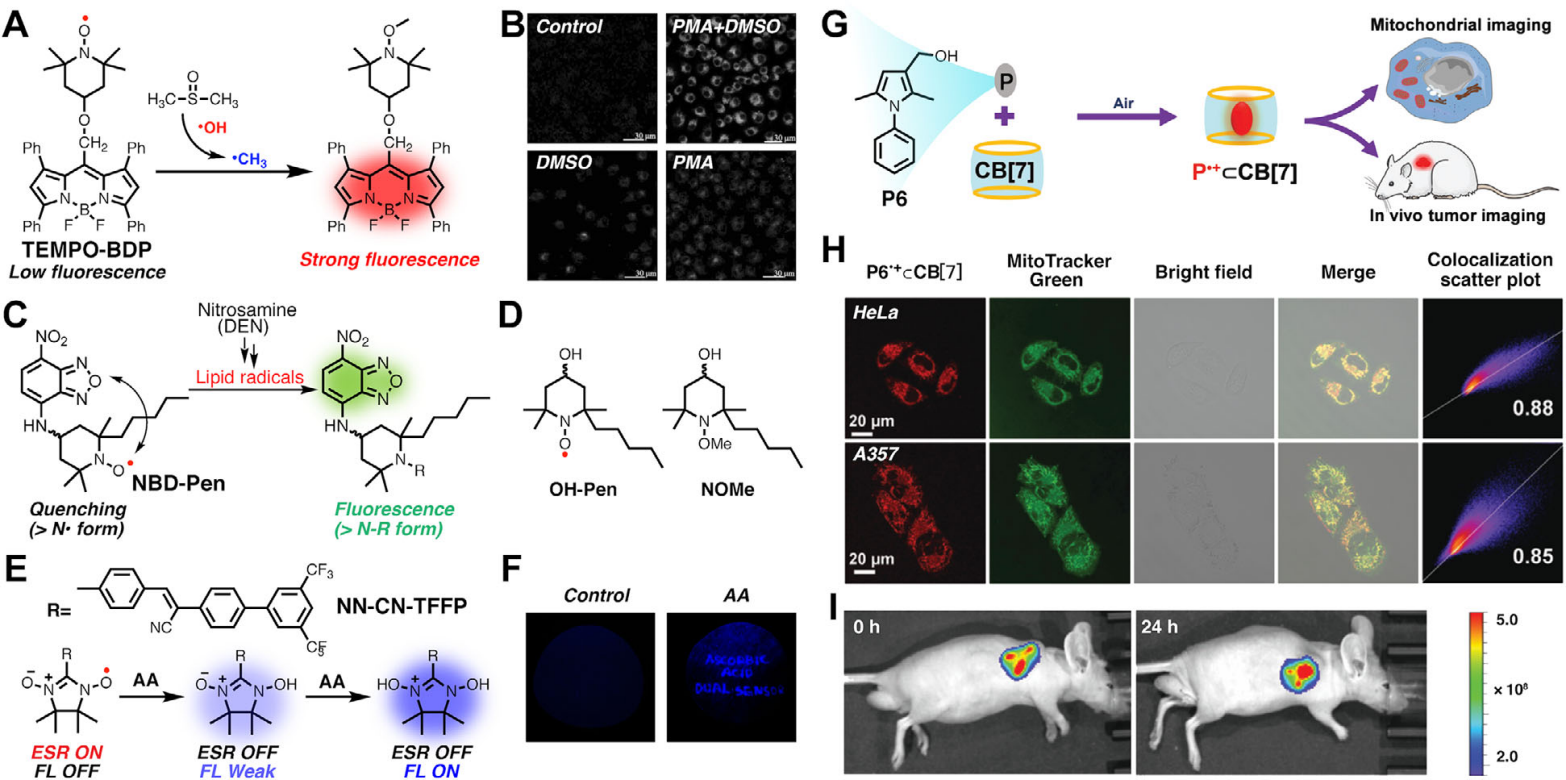
(A) Chemical structures of TEMPO‐BDP and its mechanism for hydroxyl radical detection. (B) Intracellular hydroxyl radical detection after various treatments. Reproduced with permission.^[^ ^29^ ^]^ Copyright 2010, John Wiley and Sons. (C) Chemical structures of NBD‐Pen and its mechanism for lipid radical detection. (D) Chemical structures of OH‐Pen and NOMe. (E) Chemical structures of NN‐CN‐TFFP and mechanism for detection AA. (F) Fluorescence images of a paper immersed with NN‐CN‐TFFP under 365 nm UV lamp before (left) and after (right) writing letters with AA solution. Reproduced with permission.^[^ ^31^ ^]^ Copyright 2016, American Chemical Society. (G) The preparation process of radical cations P•+ ⊂ CB[7] and its applications for mitochondrial imaging and in vivo tumor imaging. (H) Co‐localization images between P6•+ ⊂ CB[7] and MitoTracker Green in different cell lines. (I) In vivo tumor images before and after 24 h intratumoral injection of P6•+ ⊂ CB[7]. Reproduced with permission.^[^ ^32^ ^]^ Copyright 2021, Royal Society of Chemistry

Fluorescence probes have been applied as effective tools for analytical sensing and imaging. Fluorescence sensors generally rely on reversible or irreversible chemical reactions with their targeting species to produce products that can timely provide emission signals.^[^ ^27^ ^]^ Fluorescence imaging mainly involves fluorescence microscopy and fluorescent dyes, by which tissues, organs, cells, or cellular compartments can be visualized.^[^ ^28^ ^]^ They are featured with many merits such as less invasion, high spatial‐temporal resolution, real‐time monitoring, and low cost. Nitroxides have long been known for their fluorescence quenching abilities on fluorophores by annihilating their singlet excited states. Interestingly, the fluorescence can be recovered after the nitroxides were either captured by other radicals or reduced into hydroxylamines. Hence, this switchable fluorescence offers a powerful tool for mapping the redox status in tissues or organs. Tang et al. reported a radical‐fluorophore dyad (TEMPO‐BDP) by covalently attaching TEMPO to boron dipyrromethene (BDP), as shown in Figure 6A.^[^ ^29^ ^]^ The TEMPO moiety in TEMPO‐BDP can react with a methyl radical (•CH_3_) produced by the reaction between •OH and dimethylsulfoxide (DMSO). After the reaction, the product shows strong fluorescence while TEMPO‐BDP itself has low fluorescence because the fluorescence of BDP was almost quenched by TEMPO (Figure 6A). Then TEMPO‐BDP was used for imaging of •OH in DMSO/PMA (Phorobol 12‐myristate 13‐acetate)‐preincubated mice macrophages (Figure 6B). In a later report, Yamada et al. also utilized the function of nitroxide to switch fluorescence.^[^ ^30^ ^]^ They reported a fluorescence nitroxide probe (NBD‐Pen) for detecting lipid radicals, as shown in Figure 6C. Generation of lipid radicals was triggered by adding arachidonic acid (AA) and lipoxygenase (LOX) together. Slowly decreased EPR signal and enhanced fluorescence intensity were observed, confirming that the NBD‐Pen probe was a qualified lipid radical sensor (Figure 6C). Diethylnitrosamine (DEN) was administrated to induce a mice model of hepatic carcinoma, obvious lipid radical generation was observed within 1 h. Moreover, they prepared OH‐Pen with lipid radical scavenging ability and a similar molecule (NOMe) without lipid‐radical‐capturing ability for in vivo study (Figure 6D). As excepted, OH‐Pen showed much better therapeutic performances than the NOMe group. In 2016, Park et al. demonstrated an AA probe (NN‐CN‐TFFP) which is composed of a nitronyl‐nitroxide moiety and a fluorescence cyanostilbene moiety (Figure 6E).^[^ ^31^ ^]^ Upon gradual addition of AA, NN‐CN‐TFFP was reduced into its hydroxylamine form, which accompanied with decreased EPR signal and increased blue fluorescence (260‐fold). As a dual‐modes probe, NN‐CN‐TFFP also shows broad detection range and favorable selectivity over different antioxidants and acids. Moreover, a fluorescent paper for detection of AA is made by immersing a filter paper in the solution of NN‐CN‐TFFP. As shown in Figure 6F, obvious blue fluorescence can be observed after writing letters with AA ink.

### 3.5 Fluorescence imaging

Tang et al. demonstrated a series of red‐to‐near infrared (NIR) emissive carbon‐centered radical cations based on electron‐rich 2,5‐dimethylpyrroles for mitochondrial targeting and in vivo fluorescence imaging via an in situ “air oxidation method”.^[^ ^32^ ^]^ As shown in Figure 6G, upon an in situ redox reaction, a pyrrole‐based molecule (P) is encapsulated and stabilized by cucurbit[7]uril (CB[7]) to generate a supramolecular radical cation (P^•+ ^⊂ CB[7]). P6 with a 3‐hydroxylmethyl group on a pyrrole ring was shown to have a fast reaction with CB[7] to give P6^•+ ^⊂ CB[7] with efficient (quantum yield = 11.32%) fluorescence at 670 nm. Figure 6H shows red fluorescence from P6^•+ ^⊂ CB[7] in HeLa and A357 cells with locations matched well with those of MitoTracker demonstrating good mitochondria‐targeting ability of P6^•+ ^⊂ CB[7]. Moreover, the in vivo fluorescence imaging was carried out after intratumoral injection of P6^•+ ^⊂ CB[7], and its fluorescence intensity was well‐maintained 24 h post injection, confirming its good stability for long‐term monitoring (Figure 6I).

## 4 PHOTO‐TRIGGERED THERAPY

### 4.1 Photodynamic therapy (PDT)

**FIGURE 7:**
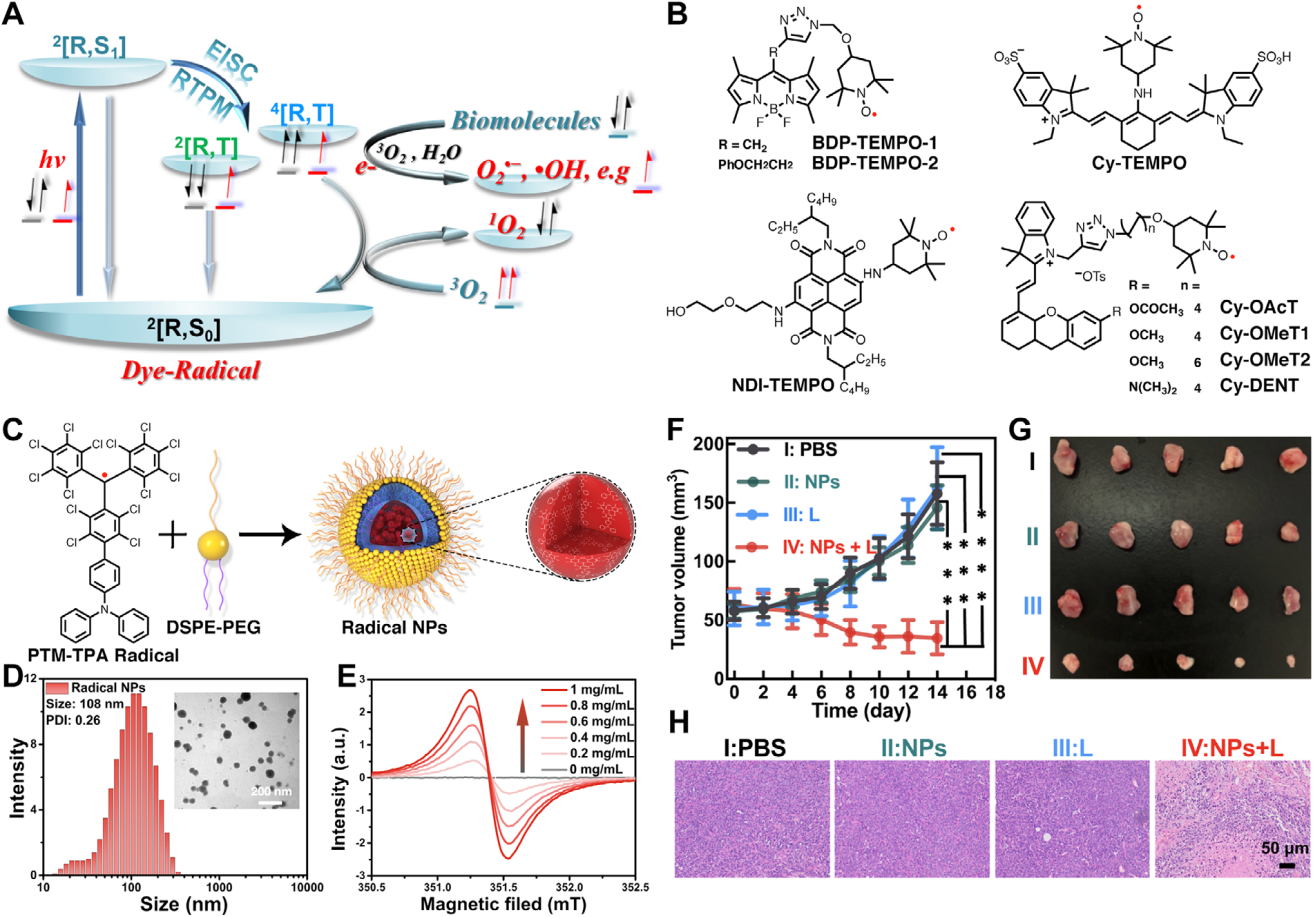
(A) Mechanisms for dye‐radical to produce toxic ROS. (B) Chemical structures of nitroxide: BDP‐TEMPO‐1, BDP‐TEMPO‐2, Cy‐TEMPO, NDI‐TEMPO, Cy‐DENT, and derivatives. (C) Chemical structures of PTM‐TPA carbon radical and radical nanoparticles preparation via nanoprecipitation method. (D) Hydrodynamic size and TEM images of radical NPs. (E) EPR spectra of radical NPs under different concentrations. (F,G) In vivo antitumor effect of nanoparticles upon 635 nm irradiation (NPs+L). (H) H&E staining of tumor slices collected from tumors. Reproduced with permission.^[^ ^38^ ^]^ Copyright 2021, Royal Society of Chemistry

PDT as an alternative modality of cancer treatment has drawn tremendous recent attractions. It mainly utilizes photosensitizers (PSs), triplet oxygen (O_2_), and light of appropriate wavelengths to produce ROS for killing cancer cells. PDT is superior to conventional treatments such as surgery, chemotherapy, and radiotherapy because it is less invasive, site‐specific, safe, and cost‐effective.^[^ ^33^ ^]^ ROS generated by most PDT processes is singlet oxygen (^1^O_2_). Such PDT is referred to as type‐II PDT whose efficacy relies on a good supply of environmental oxygen. Unfortunately, typical tumor microenvironments have low oxygen contents, and such hypoxia often limits the effectiveness of type‐II PDT. For a small portion of PSs, free radicals (e.g., O_2_^•−^, •OH, etc.) are also generated upon photoexcitation. These are referred as the type‐I PDT process which has been shown to have much lower oxygen dependency and maintain its effectiveness in hypoxia. Recently, several radical‐PSs each with an unpaired electron have been reported to have either efficient ^1^O_2_ generation or generate free radicals upon photoexcitation in hypoxia. Upon light irradiation, the ground doublet state ^2^[R, S_0_] of radical is first excited to the excited doublet state ^2^[R, S_1_] (Figure 7A). Then it undergoes intersystem crossing to form multiple spin states including triplet state ^2^[R, T] and quartet state ^4^[R, T]. ISC process of radical from ^2^[R, S_1_] to ^2^[R, T] is spin‐allowed leading to enhanced ISC (EISC). Then the ^2^[R, T] and/or ^4^[R, T] states transfer either electrons to form free radicals or transfer energy to generate ^1^O_2_, which significantly lead to oxidative stress to cells.

#### 4.1.1 Nitroxide

PSs based on nitroxides are typically fabricated by connecting TEMPO with fluorophores such as Bodipy, naphthalenediimide (NDI), and cyanine (Cy), as shown in Figure 7B. Compared to the traditional method that uses heavy atoms to enhance ISC, introducing TEMPO shows less dark cytotoxicity and high biocompatibility. In 2017, Zhao et al. reported a long‐lived triplet radical‐Bodipy by covalently linking a TEMPO moiety to Bodipy (BDP‐TEMPO). By varying the linker between TEMPO and Bodipy, they synthesized BDP‐TEMPO‐1 and BDP‐TEMPO‐2, as shown in Figure 7B.^[^ ^34^ ^]^ By recording the continous wave (CW) and time‐resolved transient (TR) optical spectroscopies/EPR, they studied the photophysical properties of BDP‐TEMPO. The results reveal that TEMPO can greatly quench Bodipy's fluorescence while triggering long‐lived triplet excited state. This work confirmed that introducing radical has profound effect on electron spin polarization, singlet excited state, lifetime, and production efficiency of triplet excited state and ISC, which lays solid foundation for the following research on radical‐based chromophores.

In 2018, Song et al. reported a stable radical photosensitizer via attaching TEMPO to the skeleton of a heptamethine aminocyanine molecule (refereed as Cy‐TEMPO, Figure 7B).^[^ ^35^ ^]^ Long‐lived triplet excited state is a key parameter for evaluating the enhanced ISC after introducing TEMPO. Hence, lifetime of triplet state was then studied by time‐resolved transient difference absorption spectroscopy. Two peaks at around 660 and 790 nm are respectively ascribed to the ground state and the triplet excited state (*τ *= 9.16 μs). Cy‐TEMPO exhibits higher ^1^O_2_ quantum yield (Φ_Δ _= 20%) than that of Cy (Φ_Δ _= 0.006%) in ethanol. Moreover, upon irradiation of a 660 nm laser, Cy‐TEMPO demonstrated an effective IC_50_ value of 50.4 μM in HeLa cells line.

Soon afterward, Li et al. demonstrated a stable radical photosensitizer (NDI‐TEMPO) by covalently linking TEMPO with NDI, as shown in Figure 7B.^[^ ^36^ ^]^ NDI‐TEMPO demonstrated high triplet state quantum yield (Φ_T _= 74 %), a long‐lived triplet state (*τ *= 8.7 μs), and fast ISC (1/K_EISC _= 338 ps), which is beneficial for improving the ^1^O_2_ generation efficiency. Strong emissive (E) polarization was firstly observed in D_0_ state of the TEMPO moiety owing to the quenching of the excited singlet state of NDI by the radical moiety. Interestingly, within 3 μs, absorptive polarization by TEMPO moiety was subsequently observed by TREPR, which was inverted from the emissive polarization. Both emissive and absorption polarization dramatically lead to the quenching of both singlet and the triplet excited state of NDI moiety. The formation and decay of a quartet state (*S* = 3/2) were observed in NDI‐TEMPO. To study its photocytotoxicity upon red‐light irradiation, NDI‐TEMPO molecule was encapsulated into liposomes, which demonstrated good biocompatibility and a low IC_50_ value of 3.22 μM in HeLa cells.

Peng et al. recently reported a series of quartet state radical photosensitizers by attaching hemicyanine to TEMPO (referred as Cy‐DENT, Figure 7B).^[^ ^37^ ^]^ After introducing the TEMPO radical, quartet state ^4^[R, T] was observed in Cy‐DENT by TREPR, and significantly results in 20 times increase of ^1^O_2_ quantum yield (*Φ*_Δ _= 32.3%) compared to corresponding molecule without TEMPO. Moreover, different substituent groups with varying electron donating ability were introduced, their influence on ^1^O_2_ quantum yield was carefully compared. In vivo PDT study was carried out in 4T1 tumor bearing mice, no obvious tumor growth was observed in the mice treated with Cy‐DENT.

#### 4.1.2 Carbon‐centered radical

Lee et al. recently demonstrated a stable carbon‐centered monoradical photosensitizer (PTM‐TPA) via conjugating a perchlorotriphenylmethyl (PTM) radical to a triphenylamine (TPA) group, as shown in Figure 7C.^[^ ^38^ ^]^ PTM‐TPA molecules were co‐assembled with DSPE‐PEG via the standard nanoprecipitation method into nanoparticles with good water dispersibility (Figure 7C and 7D). EPR spectra of the nanoparticles show representative carbon‐radical signature (Figure 7E). Upon photoexcitation in hypoxia, the nanoparticles can effectively generate O_2_^•−^. In vivo experiment using a mouse model also demonstrated effective elimination of tumor (NPs+L group in Figure 7F) and good biosafety (Figure 7H). Under the catalysis of superoxide dismutase (SOD) inside cells, a portion of O_2_^−•^ is transformed into hydrogen peroxide (H_2_O_2_) and O_2_. In the presence Fe^2+^, H_2_O_2_ will then form hydroxyl radical (•OH) which can kill cancer cells. Even though there is very little oxygen supply in the initial environment, type‐I PDT executes O_2_‐recycled pathway instead of consumed. While this is so far the only example on stable carbon‐centered radical material for PDT applications, this work clearly suggests that this class of materials does have good potential and deserves further exploration.

### 4.2 Photothermal therapy (PTT)

As a peer of PDT, PTT is also a non‐invasive therapeutic method that mainly utilizes non‐radiative decay of photoexcited agents. Different from PDT which relies on cytotoxic ROS to kill cancer cells, PTT uses in situ heating for cancer ablation. Up to now, various NIR‐I or even NIR‐II PTT agents have been developed, achieving effective in vivo efficacy. For PTT agents, desirable photo‐absorbing ability, effective photo‐thermal conversion, superior photostability, good degradability, and excellent biocompatibility are critically important.^[^ ^39^ ^]^ It is well acknowledged that open‐shell organic radical materials intrinsically have narrower energy gaps than closed‐shell non‐radical materials. In addition to their narrower energy gaps, radical materials also have higher non‐radiative decay rates. These are beneficial for optical absorption and conversion to heat, rendering them good potential as PTT agents.^[^ ^40^ ^]^

#### 4.2.1 Carbon‐centered radical

**FIGURE 8:**
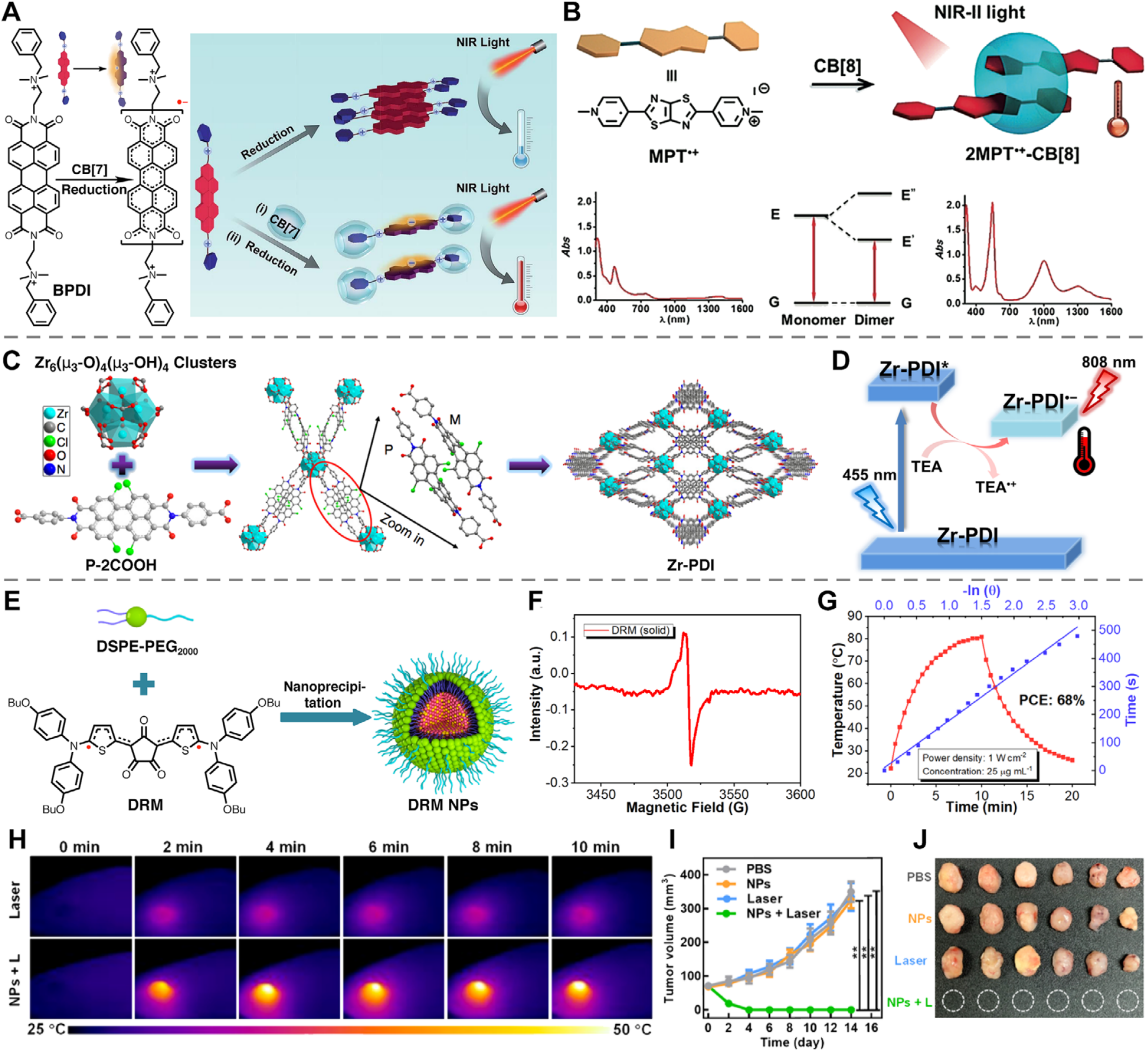
(A) Chemical structures of BPDI and BPDI radical anion after reduction and scheme illustration of different photothermal effects of BPDI radical anions without (low temperature increase) and with CB[7] (high temperature increase). Reproduced with permission.^[^ ^41^ ^]^ Copyright 2015, Royal Society of Chemistry. (B) NIR‐II photothermal effect of 2MPT•+‐CB[8] and UV/Vis‐NIR absorption of MPT•+ and 2MPT•+‐CB[8]. Reproduced with permission.^[^ ^42^ ^]^ Copyright 2019, John Wiley and Sons. (C) Structures of Zr‐cluster and P‐2COOH and the synthesis of Zr‐PDI MOF. Reproduced with permission.^[^ ^43^ ^]^ Copyright 2019, Springer Nature Limited. (D) Schematic illustration processes of Zr‐PDI* (excited state) formation after 455 nm light excitation, Zr‐PDI•− formation after reduction by TEA, and photothermal effect of Zr‐PDI•− upon irradiation of 808 nm laser. (E) Chemical structure of DRM and the preparation process of DRM NPs. (F) EPR spectra of DRM. (G) Temperature increase and decrease curves as well as linear fitting between ‐ln (θ) and time. (H) Thermal imaging of mice after different treatments (laser and NPs+L). (I,J) In vivo antitumor effect of DRM NPs. Reproduced with permission.^[^ ^45^ ^]^ Copyright 2021, American Chemical Society

Zhang et al. demonstrated a perylene diimide (PDI) anionic radical for NIR PTT. A PDI derivative (BPDI in Figure 8A) was first reduced to yield a PDI radical anion, whose edge group was then encapsulated by Cucurbit[7]uril (CB[7]) via host‐guest interactions.^[^ ^41^ ^]^ Moreover, CB[7] can also protect the PDI‐based radicals from dimerization and avoid aggregation‐induced quenching in aqueous solution. Upon encapsulation using CB[7], photothermal conversion efficiency (PCE) of BPDI under 808 nm irradiation increased from 16.3% to 31.6%. Compared with traditional radical synthesis method via covalent chemistry, the method they established via supramolecular chemistry is much simpler. Later, the same group took their work one step forward by developing a radical dimer (2MPT^•+^‐CB[8]) with NIR‐II absorption (Figure 8B).^[^ ^42^ ^]^ It was fabricated by cationic carbon‐centered radical (MPT^•+^) and cucurbit[8]uril (CB[8]). 2MPT^•+^‐CB[8] demonstrated considerably higher PCE (54.6%) and obvious cytotoxicity toward HepG 2 cancer cells upon irradiation of a 1064 nm laser.

Yin et al. reported a novel PDI anionic radical (Zr‐PDI^•−^), which shows excellent stability and desirable NIR absorption.^[^ ^43^ ^]^ As shown in Figure 8C, a Zr‐PDI metal organic framework (MOF) was fabricated by coordination between N, N’‐di‐(4‐benzoic acid)‐1,2,6,7‐tetrachloro‐perylene‐3,4,9,10‐tetracarboxylic acid diimide (P‐2COOH) ligand and Zirconium (Zr^4+^). Upon irradiation of blue light (455 nm) and exposure to vapor of triethylamine (TEA), Zr‐PDI got electron from TEA to form anionic Zr‐PDI^•−^ (Figure 8D) via photo‐induced electron transfer (PET). The obtained Zr‐PDI^•−^ shows desirable NIR absorption and a high PCE of 52.3%.

#### 4.2.2 Carbon‐centered diradical

Recently, Tang et al. reported photothermal performances of a stable delocalized carbon‐centered diradical (CR‐TPE‐T) for water evaporation.^[^ ^44^ ^]^ The broad absorption (300–1600 nm) endows CR‐TPE‐T with excellent light harvesting ability and high PCE (72.7%) upon 808 nm laser irradiation. Lee et al. also exploit a novel carbon‐centered diradical DRM, which is composed of a strong donor (N,N‐bis(4‐butoxyphenyl)thiophen‐2‐amine) and a robust acceptor (croconic acid), as shown in Figure 8E.^[^ ^45^ ^]^ Obvious feature of radical was observed in EPR spectra of the DRM because of efficient charge transfer via interaction between donor and acceptor (Figure 8F). After assembling DRM into nanoparticles (Figure 8E), DRM NPs show desirable absorption at∼790 nm, which is highly favorable for NIR PTT. Significantly, DRM NPs exhibit superior light‐harvesting ability (∼220 L g^–1^ cm^–1^) and a PCE of 68% (Figure 8G). Finally, photothermal performance and antitumor effect of DRM NPs were studied. Obvious temperature increase (Figure 8H) and excellent antitumor performances were observed in vivo (Figure 8I,J).

## 5 SUMMARY AND OUTLOOKS

The unique spin, magnetics, optics, redox properties of radicals, and their superiorities over conventional closed‐shell materials are discussed in this perspective. Furthermore, we summarized recent development of these radical materials and their biomedical applications for imaging and sensing, and photo‐triggered therapies. However, the development of radical materials and their biological applications are still in their early development stage. A short discussion of the challenges and outlooks of these radical materials is thus given here.

1. Stable organic radical materials with high relaxivity for T_1_ and/or T_2_ in MRI are still rare and this severely hinders their practical applications. The intrinsic low unpaired electron of nitroxide often leads to low relaxivities compared to metal‐based Gd^3+^ (with seven unpaired electrons) and Mn^2+^ (five unpaired electrons). Radicals can also be removed by fast reduction in biological environments, thus rendering them ineffective contrast agents. On the one hand, increasing spin concentrations can be resorted to enhance relaxivities of radicals via extension of skeletons. On the other hand, the high‐spin radicals (≥2 unpaired electrons) are highly anticipated to develop. Moreover, water solubility/dispersibility and stability should also be considered when designing practical high performance ORCAs.
2. Radical materials have been recognized as efficient molecular tools for sensing/imaging and therapies in biological systems. However, the emission/absorption of most reported radical materials is mainly located in the UV or visible region. Developing radical materials with NIR‐I/II emission/absorption are highly preferred owing to reduced interferences of background absorption, higher tissue penetration depth, and less harmful to body tissues. Moreover, most reported radical materials do not have intrinsic ability for targeting biomolecules of interest. This is certainly an area that deserves more exploration for effective ways for decorating identifiable ligands which can offer anchoring effect toward biomolecules. Finally, it has been shown that working mechanism of type‐I PDT is less dependent on the oxygen level, which is highly favorable for hypoxia‐overcoming therapies toward deeper solid tumors. More efforts should be devoted to exploit radical materials with NIR‐absorption for type‐I PDT.
3. The development of radical discipline should not only expand their new applications, but also boost the collaboration of more than two functions in a single molecule. For example, MRI offers high tissue penetration depth, three‐dimensional spatial resolution, and fluorescence imaging enables high sensitivity and real‐time monitoring. Meanwhile, radical materials are potential candidates for highly effective type‐II PDT or hypoxia‐overcoming type‐I PDT. It is highly urgent to explore entirely organic radical materials for dual‐modality MRI/fluorescence imaging and type‐I or II PDT, demonstrating potential for molecular theranostic applications.
4. Radical materials for biosensing or bioimaging are in an always‐on mode, thus, it is imperative for the development of smart (response, excitation, and switch) systems regulated by physic or chemistry (thermal, ultrasound, light, magnetic, pH, enzymes, redox, ROS, biomolecules).
5. From the view of molecular designs, stability and spin‐spin interactions of radicals should be well balanced and carefully considered to promote the developments of radical materials. Although there are considerably numerous stable radicals, their biological applications are mainly limited to carbon‐centered radicals and nitroxides. The biological applications of stable radical materials based on silicon, germanium, beryllium, and boryl centers/groups are less reported and should be widely exploited.

## CONFLICT OF INTEREST

The authors declare that there is no conflict of interest.
